# H_2_TPP Organocatalysis in Mild and Highly Regioselective Ring Opening of Epoxides to Halo Alcohols by Means of Halogen Elements

**DOI:** 10.3390/molecules17055508

**Published:** 2012-05-09

**Authors:** Parviz Torabi, Javad Azizian, Shahab Zomorodbakhsh

**Affiliations:** 1 Department of Chemistry, Islamic Azad University, Mahshahr Branch, Mahshahr 63519, Iran; 2 Department of Chemistry, Faculty of Science, Science and Research Branch, Islamic Azad University, Tehran 11365, Iran

**Keywords:** oxirane, ring opening, nucleophilic addition, elemental halogen, *meso*-tetraarylporphyrine, halohydrine

## Abstract

We found that elemental iodine and bromine are converted to trihalide nucleophiles (triiodine and tribromide anion, respectively) in the presence of catalytic amounts of *meso*-tetraphenylporphyrins (H_2_TPP). Therefore a highly regioselective method for the synthesis of β-haloalcohols through direct ring opening of epoxides with elemental iodine and bromine in the presence of H_2_TPPs as new catalysts is described. At room temperature a series of epoxide derivatives were converted into the corresponding halohydrins resulting from an attack of trihalide species anion atoms at the less substituted carbon atom. This method occurs under neutral and mild conditions with high yields in various aprotic solvents, even when sensitive functional groups are present.

## 1. Introduction

Oxiranes are among the most versatile intermediates in organic synthesis, as they can be easily prepared from a variety of other functional groups [1] and due to their ring strain and high reactivity, their reactions with various nucleophiles lead to highly regio and stereoselective ring opened products [2,3,4]. Vicinal halohydrins have found wide applications in organic transformations and in the synthesis of marine natural products [5,6]. The availability of some epoxides in an optically active form has enhanced their use as synthetic intermediates; a reaction sequence allows an impressive access to a large variety of compounds in an optically active form [7,8]. However, their direct conversion to halohydrins remains a reaction of considerable interest [9].

A variety of reagents are known to convert epoxides to halohydrins; the ring openings of unsymmetrically substituted epoxides with Li_2_(NiBr_4_) [10], LiX-(Bmim)PF_6_ [11], haloborane reagents [12], Br_2_/PPh_3_ [13], SmI_2_ [14], Ti(O-i-pr)_4_ [15], chlorosilanes [16], Lewis acids [3,17,18] and BF_3_-Et_2_O [19] have been reported. In particular metal halides such as Li/Ti [2], Sn [20], P [13], Cu [21], and Ni [10] easily induce epoxide-opening, in which the use of a stronger Lewis acid and a metal ion in structure of catalyst often results in low yields of the halohydrins when other sensitive functional groups are present [22]. However, in these approaches we encountered with some limitations, such as the need for strong Lewis acid and protic media that certainly are unsuitable conditions for complex epoxide compounds.

Recently, it has been found that epoxides can be converted into iodoalcohols and bromoalcohols by elemental iodine and bromine, in the presence of some specific compounds such as Mn(II) salen complexes [23], 2-phenyl-2-(2-pyridyl)imidazolidine [24], thiourea [25] and diamines [26] as efficient catalysts. Among these catalysts, the Mn(II) salen complexes are more efficient and effective, but in this method, the oxidation of metal(II) in complex catalyst is an important limitation and reduced the activity of catalyst for next reusability.

We would like to describe herein that H_2_TPP’s are highly reactive catalysts for the cleavage of epoxide rings to relative vicinal halohydrins in the presence of elemental iodine and bromine, more efficiently and regioselectively and in high yield under mild conditions that are highly desirable. The catalysts are easily recovered and can be reused several times.

## 2. Results and Discussion

**Scheme 1:**
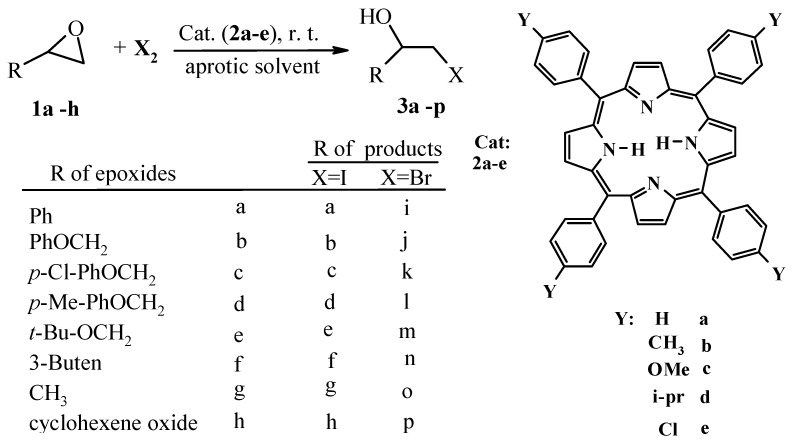
Catalytic conversion of epoxides to halohydrins.

In this study, the reaction of styrene oxide with iodine and bromine in the presence of some derivatives of H_2_TPP as the catalyst were carried out (Scheme 1).

Derivatives of H_2_TPP and metal-TPP’s have been recognized as being among the most promising catalysts for various reactions [27,28]. These compounds show wide applicability and are now used as catalysts for a variety of regio and enantioselective reactions, such as CO_2_/epoxide coupling [29], acetolysis, hydrolysis and alcoholysis [30]. In all of these transformations, the coordinated metal ion in catalyst has a key role in the reaction process and this necessity causes some destruction of sensitive functional groups. After a solution of styrene oxide and a catalyst in CH_2_Cl_2_ was stirred in room temperature, a solution of elemental halogen in CH_2_Cl_2_ was added dropwise. The amount of the catalyst was a 0.05 molar amount of the styrene oxide used. The reaction product was 2-halo-1-phenylethanol (**3a**, **3i**), and the yield was determined by GC analysis (Table 1). In each case, cleavage of the epoxide ring occurs and, upon thiosulphate workup, iodo- and bromoalcohol are obtained. The catalysts are easily recovered and can be reused several times. Tetraphenylporphyrin derivatives were prepared and metallated according to the literature [31,32].

To ascertain the scope and limitation of the present reaction, a wide range of structurally diverse epoxides were subjected to cleavage by this method to produce the corresponding halohydrins. These results are summarized in Table 2. For comparison, a number of methods for the conversion of oxiranes to the corresponding halo alcohols are given in entries 10–14 (Table 2).

However, other factors can exert a controlling influence such as: (1) steric hindrance of the epoxides (for example, compare in Table 2, entry 7 with entry 8); (2) the nature of the solvent; (3) the rate of admixing the reagents; and (4) the order in which the reagents are combined. Each one can have a pronounced effect on the observed ratio of β-halohydrin isomers and the overall yield.

The order and rate in which the reagents are combined were found to exert a subtle influence on the yield and regioselectivity in both bromohydrin and iodohydrin formation. However, if bromine is added to the epoxide before the catalyst, two isomeric bromoalcohols are produced, but if the epoxide is added to catalyst and then bromine is added dropwise over a period of time, only one isomer is formed. Furthermore, the rapid addition of bromine reduced the regioselectivity.

The results of the ring opening of styrene oxide in the presence of catalyst **2a** in various solvents are summarized in Table 3. The iodination and bromination reactions can cleanly proceed in dichloromethane, while those performed in THF, DMSO, chloroform, diethylether and acetonitrile lead to a lower yield of the β-halohydrins. Thus, these reactions appeared to be heavily dependent on the nature of the solvent.

**Scheme 2:**
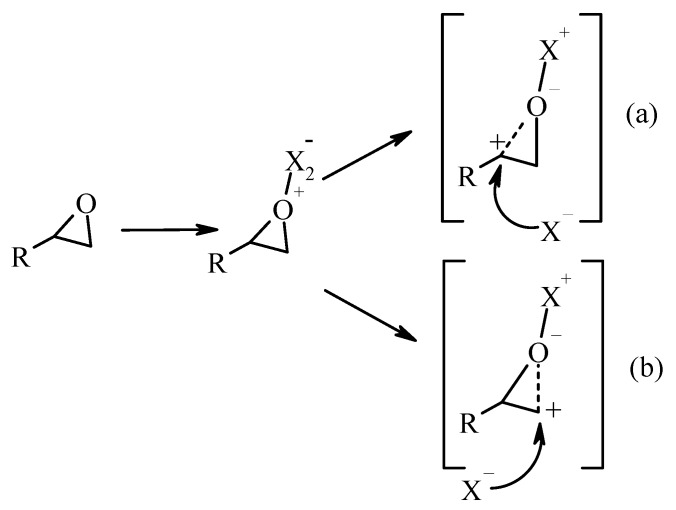
Tow possible way for nucleophilic ring opening.

As shown in Table 2, an anti Markovnikov-type regioselectively [34] is generally observed in these reactions. An attack of the nucleophile preferentially occurs at the less-substituted oxirane carbon atom that this type of regioselectively appears to be the opposite of that observed in ring opening of the same epoxides with aqueous hydrogen halides under classic acidic conditions [29] (entry 13, Table 2). The cyclic epoxides (entries 5, 18) always produced *trans*-halohydrins as indicated by the observed coupling constants of the ring of the hydrogens in their ^1^H-NMR spectra. When catalyst is not present, the cleavage of epoxides can occur via two limiting mechanistic pathways, either an electrophilic attack by halogen, behaving as a Lewis acid, giving the more-stable carbenium ion-like transition state a, or via nucleophilic attack by a halide ion on the epoxide-halogen complex, giving the more stable transition state b (Scheme 2). However, this new method appears to be highly competitive with the other methods reported in the literature. The reaction occurs under neutral and mild conditions on the acid sensitive substrates and vicinal halohydrins were obtained in high yields and with high regioselectivity.

**Figure 1:**
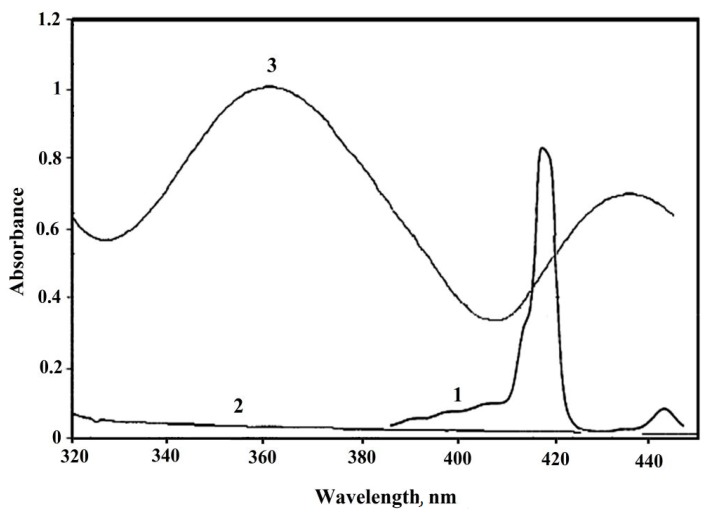
Absorption Spectra of: (**1**) Catalyst 2a; (**2**) Iodine (**3**) Catalyst 2a:I_2_ with Molar ratio 0.2:1 in Dichloromethane Solution.

**Figure 2:**
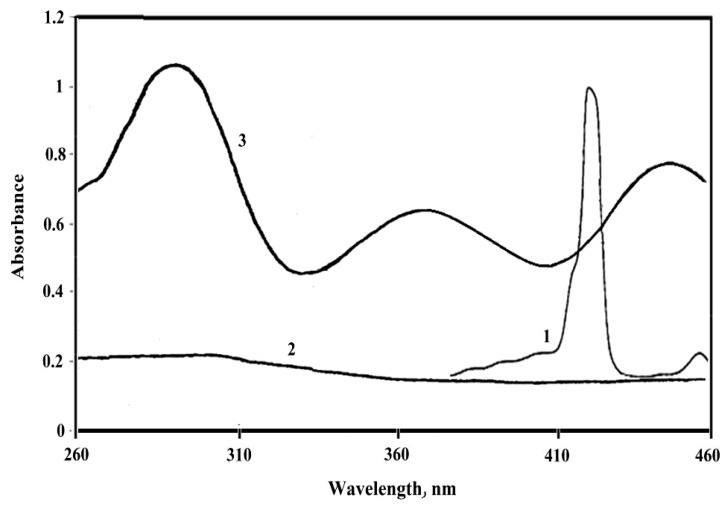
Absorption Spectra of: (**1**) Catalyst 2a; (**2**) Bromine (**3**) Catalyst 2a:Br_2_ with Molar ratio 0.2:1 in Dichloromethane Solution.

Based on our study on the complexation of porphyrins and other works reported on the different compounds with elemental halogen [23,24,26], it seems that halogenative cleavage of epoxides occurs via trihalide ion, X_3_^−^ as the nucleophile. In support of this suggestion, the electronic absorption spectra of catalyst (1), iodine (2) and complex formation between iodine and bromine in the presence of 2a as catalyst (3) in dichloromethane solution at 25 °C are shown in Figure 1 and Figure 2.

**Scheme 3:**
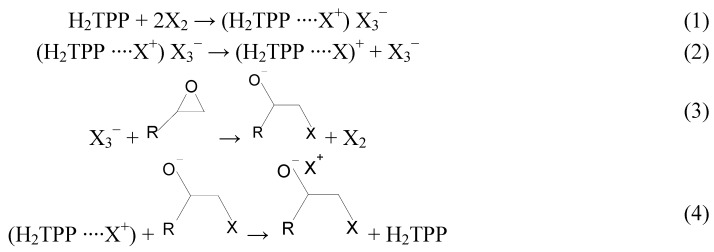
A four-step mechanism for halogenative cleavage of epoxide.

The electronic absorption spectra of the related addition of H_2_TPP to iodine and bromine has shown strong absorption band at 365 nm for I_2_ addition and 272 nm for Br_2_ addition (Figure 1 and Figure 2) respectively, presumably due to the complex formation of this ligand with I_2_ and Br_2_. It should be noted that the bands of 364 nm and 272 nm are characteristic of the formation of I_3_^−^ and Br_3_^−^ ions, respectively [35,36], in the process of complex formation of different electron-pair donor ligands with iodine and bromine, while none of the initial reactants show any measurable absorption in these regions. Thus we suggested a four-step mechanism for halogenative cleavage of epoxides in the presence of catalytic amounts of porphyrin:

The first step [Equation (1), Scheme 3] involves the formation of a 1:2 or 1:1 molecular complex between the catalyst and elemental halogen, in which the halogen ion (X_3_^−^) exists as a contact ion pair. In the second step [Equation (2)] this complex is further decomposed to release the X_3_^−^ nucleophile ion into the solution. Therefore, in this way, molecular iodine or bromine is converted to a nucleophilic halogen species in the presence of H_2_TPP and, in the third step Equations (3) and (4), this ion participates in the ring opening reaction of the epoxides, and the catalyst is reproduced and is used in the first step again. These steps occur continuously until all of the epoxides and halogen are consumed, however, in each case, cleavage of the epoxide ring occurs, after work-up with thiosulfate the catalyst can be easily recovered and could be reused several times. One of the advantage of H_2_TPP as catalyst for promoted the elemental halogen in the ring opening of epoxides, is activation of nucleophile without any metal ion, and therefore the activity of catalyst is constant, and also in reusability of the catalyst, we observed a negligible decrease of the activity.

The reusability of catalyst was tested in the reaction of styrene oxide with I_2_ in the presence of the catalytic amounts of H_2_TPP in CH_2_Cl_2_ media at room temperature (Table 4). After the reaction was completed, the reaction mixture was worked up as mentioned in the Experimental section; the resultant solid containing the catalyst was reused with a fresh charge of epoxide and iodine. H_2_TPP reused catalyst exhibited good activity rather than that in its original use even after five reuses. Yields of related iodohydrin in each process were decreased slowly because the amounts of the catalyst reused after every run is trifling decreased.

## 3. Experimental 

### 3.1. Materials

Chemicals were purchased from the Merck Chemical Company in high purity. All of the materials were of commercial reagent grade. The epoxides and used solvents were purified by standard procedures.

### 3.2. Apparatus

IR spectra were recorded as KBr pellets on a Perkin-Elmer 781 Spectrophotometer and an Impact 400 Nickolet FTIR Spectrophotometer (Tehran, Iran). ^1^H-NMR and ^13^C-NMR spectra were recorded in d_6_-DMSO on a Bruker DRX-400 spectrometer (Tehran, Iran) for samples as indicated with tetramethylsilane as internal reference. Mass spectra were recorded on a Finnigan MAT 44S (Tehran, Iran), by Electron Ionization (EI) mode with an ionization voltage of 70 eV. Melting points were obtained with a Yanagimoto micro melting point apparatus (Tehran, Iran) and are uncorrected. The purity determination of the substrates and reactions monitoring by the solvent system were accomplished by TLC on Polygram SILG/UV 254 silica-gel plates (from Merck Company, Tehran, Iran).

### 3.3. General Procedure for Conversion of Epoxides to β-Halohydrins

Epoxide (1 mmol) in CH_2_Cl_2_ (5 mL) was added to a stirred solution of catalyst (0.1 mmol) in CH_2_Cl_2_ (5 mL) at room temperature. Then a solution of elemental halogen (1 mmol) in CH_2_Cl_2_ (5 mL) was added dropwise (10 min) to the above-mentioned mixture. The progress of the reaction was monitored by TLC analysis. After complete disappearance of the starting material, the reaction was poured to the 10% aqueous Na_2_S_2_O_3_ (20 mL) and extracted with CH_2_Cl_2_ (3 × 10 mL). After the evaporation of CH_2_Cl_2_ under vacuum, the organic phase has been dried on anhydrous sodium sulphate. The product was purified by a short column chromatography through silicagel using CCl_4_/Et_2_O/EtOH (3:1:1) as eluent (note: at room temperature the catalyst is nearly insoluble in Et_2_O/EtOH mixture. Therefore, the catalyst has been washed easily from the column using CH_2_Cl_2_ as eluent). The halo alcohols were identified by a comparison with authentic samples prepared in accordance with literature procedures [15,23,26,29].

*2-Iodo-1-phenylethanol* (**3a**). IR (neat): 748 (m), 915 (m), 1032 (s), 1121 (w), 1243 (s), 1365 (m), 1492 (m), 1602 (s), 2885 (m), 2930 (s), 3061 (m), 3398 (br s) cm^−1^. ^1^H-NMR: δ = 2.02 (s, 1 H), 3.76 (d, 2 H, *J* *=* 5.5 Hz), 4.78 (t, 1 H, *J* *=* 5.0 Hz), 7.17−7.35 (m, 5 H). ^13^C-NMR: δ = 54.96, 66.90, 128.22, 129.10, 129.21, 138.17.

*1-Iodo-3-phenoxy-2-propanol* (**3b**). IR (neat): 650 (w), 678 (w), 760 (m), 823 (m), 1038 (s), 1113 (w), 1240 (s), 1375 (m), 1494 (s), 1588 (s), 2877 (m), 2927 (s), 3050 (m), 3418 (br s) cm^−1^. ^1^H-NMR: δ = 3.1 (s, 1 H), 3.48 (d, 2 H, *J* *=* 5.0 Hz), 4.06 (tt, 1 H, *J*_1_ = 7.0, *J*_2_ = 5.0 Hz), 4.13 (d, 2 H, *J* *=* 5.6 Hz), 6.78−6.90 (m, 3 H), 7.36 (m, 2 H). ^13^C-NMR: δ = 67.18, 69.67, 70.01, 114.98, 116.87, 121.79, 129.89, 132.86.

*1-Iodo-2-octanol* (**3f**). IR (neat): 725 (m), 1015 (br s), 1105 (m), 1130 (m), 1185 (s), 1385 (s), 1425 (s), 1465 (s), 1475 (s), 2870 (vs), 2940 (vs), 3400 (br s) cm^−1^. ^1^H-NMR: δ = 0.89 (t, 3 H, *J* = 7.0 Hz), 1.26−1.58 (m, 10 H), 2.24 (s, 1 H), 3.24−3.55 (m, 3 H). ^13^C-NMR: δ = 14.09, 16.45, 22.62, 25.56, 29.12, 31.70, 36.89, 70.91.

*2-Iodocyclohexanol* (**3h**). IR (neat): 690 (s), 790 (w), 870 (m), 948 (s), 1038 (w), 1082 (br s), 1123 (m), 1189 (s), 1372 (m), 1462 (s), 2882 (s), 2960 (br s), 3425 (br s) cm^−1^. ^1^H-NMR: δ = 1.26−1.44 (m, 3 H), 1.75−1.95 (m, 3 H), 2.15−2.3 (m, 1 H), 2.3−2.35 (m, 1 H), 2.72 (s, 1 H), 3.58−3.62 (m, 1 H), 3.9−4.0 (m, 1 H). ^13^C-NMR: δ = 24.51, 26.56, 32.75, 35.40, 59.84, 71, 59.

*2-Bromo-1-phenylethanol* (**3i**). IR (neat): 689 (m), 766 (m), 823 (m), 1036 (s), 1115 (w), 1233 (s), 1375 (m), 1494 (m), 1600 (s), 2875 (m), 2935 (s), 3064 (m), 3405 (br s) cm^−1^. ^1^H-NMR: δ = 1.98 (s, 1 H), 4.01 (m, 2 H), 4.98 (t, 1 H, *J* *=* 5.0 Hz), 7.19−7.39 (m, 5 H). ^13^C-NMR: δ = 57.39, 67.97, 128.32, 129.30, 129.37, 138.98.

*1-Bromo-3-phenoxy-2-propanol* (**3j**). IR (neat): 641 (w), 688 (m), 756 (m), 823 (m), 1038 (s), 1112 (w), 1239 (s), 1375 (m), 1494 (s), 1588 (s), 2878 (m), 2925 (s), 3059 (m), 3415 (br s) cm^−1^. ^1^H-NMR: δ = 2.75 (s, 1 H), 3.61 (d, 2 H, *J* *=* 5.3 Hz), 4.03 (tt, 1 H, *J*_1_ = 7.1 Hz, *J*_2_ = 5.0 Hz), 4.11 (d, 2 H, *J* *=* 7.0 Hz), 6.78 (d, 1 H, *J* = 5.0 Hz), 6.94 (d, 2 H, *J* = 8.0 Hz), 7.35 (m, 2 H). ^13^C-NMR: δ = 69.58, 69.77, 69.93, 115.01, 116.82, 121.86, 129.99, 132.79.

*1-Bromo-2-octanol* (**3n**). IR (neat): 720 (m), 830 (m), 1050 (s), 1075 (s), 1125 (m), 1225 (m), 1265 (m), 1385 (m), 1425 (m), 1470 (s), 2860 (vs), 2935 (vs), 2970 (vs) 3380 (br s) cm^−1^. ^1^H-NMR: δ = 0.89 (t, 3 H, *J* = 6.5 Hz), 1.25−1.63 (m, 8 H), 1.86 (q, 2 H, *J* = 7.1 Hz), 2.22 (s, 1 H), 3.42 (d, 2 H, *J* = 7.1 Hz), 3.75−3.84 (m, 1 H). ^13^C-NMR: δ= 14.01, 22.52, 25.58, 29.14, 31.68, 35.05, 40.73, 71.02.

*2-Bromocyclohexanol* (**3p**). IR (neat): 690 (s), 793 (w), 865 (m), 960 (s), 1038 (m), 1075 (br s), 1123 (m), 1189 (s), 1372 (m), 1460 (s), 2882 (s), 2960 (br s), 3425 (br s) cm^−1^. ^1^H-NMR: δ = 1.26−1.42 (m, 3 H), 1.78−1.98 (m, 3 H), 2.18−2.32 (m, 1 H), 2.32−2.38 (m, 1 H), 2.68 (s, 1 H), 3.58−3.64 (m, 1 H), 3.82−3.92 (m, 1 H). ^13^C-NMR: δ = 24.48, 27.02, 33.95, 36.59, 62.13, 75.66.

## 4. Conclusions

In conclusion, we have found that epoxides are cleaved regioselectively to vicinal haloalcohols under neutral conditions by elemental halogens in the presence of *meso*-tetraphenylporphyrins as catalyst. The products are obtained in high yields and after short reaction times relative to other procedures; fiurthermore, this methodology can be applied for acid sensitive substrates in aprotic solvents.

## Figures and Tables

**Table 1:** Addition of Iodine (1 mmol) and Bromine (1 mmol) to Styrene Oxide (1 mmol) in the Presence of Various Catalysts in CH_2_Cl_2_ at 25 °C.

Entry	Catalyst	Iodination		Bromination	
		Time /h	Yield ^a^ /%	Time /h	Yield ^a^ /%
1	2a	2.1	>95	1.7	>95
2	2b	2.1	90	1.7	>95
3	2c	2.2	87	1.8	90
4	2d	2.3	88	2.1	82
5	2e	2.5	76	2.0	75
6 ^**b**^	-	Several days	0	1	31 ^**c**^

**^a^** GC yield, based on epoxide; **^b^** In the presence of excess of halogen [29]; **^c^** The only one isomer, 2-bromo-2-phenyl-ethanol was formed.

**Table 2:** Reaction of various epoxides with elemental I_2_ and Br_2_ in the presence of catalyst 2a.

Entry	Epoxide (1a–h)	Conditions	Time/h	Yield ^a^ /%	Product (s) (3a–p)
1	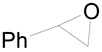	I_2_, 2a, r.t., CH_2_Cl_2_	2.1	81	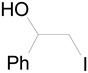
2	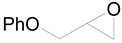	״	3.9	80	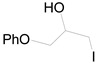
3	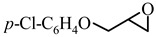	״	4.3	81	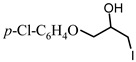
4	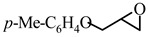	״	4.5	82	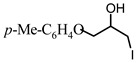
5	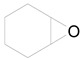	״	6.5	78	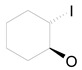
6	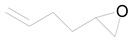	״	4.6	82	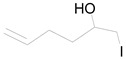
7	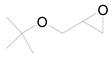	״	5.8	77	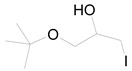
9	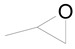	״	3.5	72	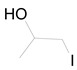
10 ^**b**^	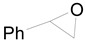	I_2_, r.t., acetone	2	83	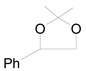
11 ^**c**^	״	[n-Bu_4_N]Br/Mg(NO_3_)_2_, CHCl_3_	5	78 (5:1)	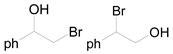
12 ^**d**^	״	(Me_2_N)_2_BBr/CH_2_Cl_2_,N_2_ atm.	12	75 (1:4.5)	״
13^**e**^	״	SmI_2_ (2 eq.), THF, −78 °C	>5 min	93	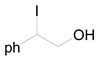
14 ^**f**^	״	NH4^+^X^−^/M^+^., CH_3_CN	1.3	87 (1:2)	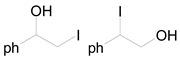
15	״	Br_2_, 2a, r.t., CH_2_Cl_2_	1.7	91	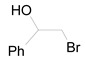
16	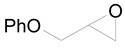	״	2.0	84	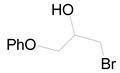
17	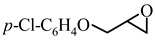	״	2.4	82	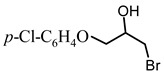
18	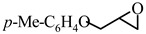	״	2.8	83	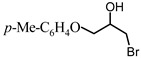
19	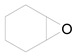	״	2.7	80	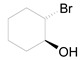
20	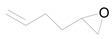	״	2.5	76	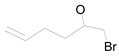
21	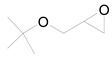	״	3.5	78	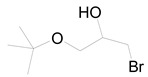
22	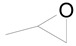	״	2.2	73	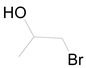

**^a^** Isolated products yields based on epoxide; **^b^** Ref. [29]; **^c^** Ref. [17]; **^d^** Ref. [33]; **^e^** Ref. [14]; **^f^** Ref. [34].

**Table 3:** Halogenative Reaction of Styrene Oxide in the Presence of Catalyst 2a in Various Solvents at 25 °C.

Entry	Solvent	Iodination		Bromination	
		Time /h	Yield ^a^ /%	Time /h	Yield /%
1	CH_2_Cl_2_	2.1	>95	1.7	>95
2	CHCl_3_	2.2	93	2.0	95
3	CH_3_CN	2.8	90	2.5	91
4	DMSO	3.2	85	3.0	88
5	THF	3.5	83	3.2	85
6	Diethyl ether	3.5	83	3.2	85

**^a^** GC Yield.

**Table 4:** Reusability of Catalyst ^a^.

Entry	Run	Time (h)	Yield (%) ^b^
1	0	2.1	>95
2	1	2.1	91
3	2	2.1	88
4	3	2.1	82
5	4	2.1	77

^a^ Addition of 1 mmol iodine to 1 mmol styrene oxide in the presence of the catalyst 2a in CH_2_Cl_2_ at 25 °C; ^b^ Based on GC yields.
